# Supplementary material for: To Close or to Collapse: The Role of Charges on Membrane Stability upon Pore Formation
Source: Adv Sci (Weinh). 2021 Mar 8;8(11):2004068. doi: 10.1002/advs.202004068 (PMC8188222; doi:10.1002/advs.202004068)
Supplement: Supplementary file 1 — Supporting Information [file ADVS-8-2004068-s001.pdf]

© 2021 Wiley-VCH GmbH

ADVANCED SCIENCE
Open Access

Supporting Information

for *Adv. Sci.,* DOI: 10.1002/advs.202004068

To close or to collapse: the role of charges on membrane stability upon pore formation

Rafael B. Lira[#], Fernanda S. C. Leomil, Renan J. Melo, Karin A. Riske, and Rumiana Dimova**

© Copyright 2020. WILEY-VCH GmbH.

Supporting Information

To close or to collapse: the role of charges on membrane stability upon pore formation

Rafael B. Lira[1,2#], Fernanda S. C. Leomil[1], Renan J. Melo[3], Karin A. Riske[1,] and Rumiana Dimova[2]**

[1]Departamento de Biofísica, Universidade Federal de São Paulo, São Paulo, Brazil, [2]Department of Theory and Biosystems, Max Planck Institute of Colloids and Interfaces, Potsdam, Germany, and [3]Instituto de Física, Universidade de São Paulo, São Paulo, Brazil

#Current address: Moleculaire Biofysica, Zernike Instituut, Rijksuniversiteit, Groningen, the Netherlands

* Address correspondence to: kariske@unifesp.br, rumiana.dimova@mpikg.mpg.de

Text S1. Electroporation conditions

The transmembrane potential built during the pulse is given by $\Psi(t) = \frac{3}{2}RE|\cos\theta|\left[1 - \exp\left(-\frac{t}{t_c}\right)\right]$ [1], where R is the vesicle radius, E is the field strength, θ is the tilt angle between the electric field and the surface normal, t is time and $t_c = RC_{me}\left(\frac{1}{\sigma_{in}} + \frac{1}{2\sigma_{ex}}\right)$ is the charging time with the membrane capacitance C_{me} and the conductivities of the solutions inside and outside the vesicle, σ_{in} and σ_{ex}, respectively. Above the electroporation threshold, the transmembrane potential Ψ cannot be further increased and the membrane becomes permeable to ions at a certain critical transmembrane potential, $\Psi_c \approx$ 1V; see e.g. [2]. In the experimental conditions explored in this work, $\sigma_{in} \cong$ 10 μS/cm, $\sigma_{ex} \cong$ 4–300 μS/cm (see also Table S1), $C_{me} \cong$ 0.01 F/m^2, the transmembrane potential reaches Ψ_c for vesicles with radii above 7–10 μm.

Table S1. Conductivity values of the solutions used. Values were obtained from three individual measurements of each solution from a single batch using a conductivity meter SevenEasy (Mettler Toledo, Switzerland).

Medium	Conductivity (μS/cm)
**Water**	1.5 ± 0.1
**Sucrose (200 mM)**	10.1 ± 0.2
**Glucose (200 mM)**	3.9 ± 0.1
**Glucose + 1 mM NaCl**	177.0 ± 1.7
**Glucose + 1 mM EDTA**	272.3 ± 1.1
**Glucose + 0.5 mM $CaCl_2$**	277.7 ± 0.6

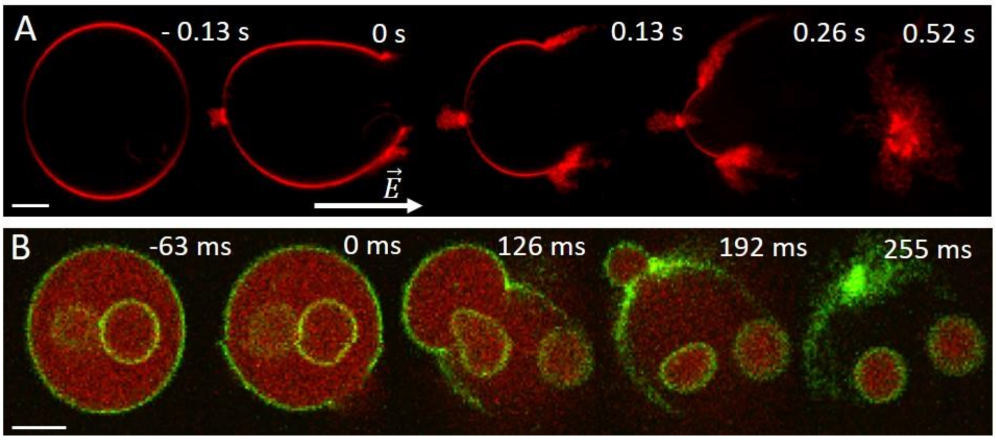

Figure S1. Bursting of charged GUVs (POPC:POPG; 1:1 mol) as observed in confocal microscopy cross-sections; the field direction is indicated by an arrow. (A) Upon bursting, the vesicle membrane around the pore rim is transformed into tubular structures whereby the tubulation is more pronounced at the vesicle pole facing the cathode. (B) Bursting results in fast and complete release of encapsulated content (internal vesicles and encapsulated SRB, green) from the GUVs; the same sequence is displayed in Movie S2. Scale bars: 10 μm. In (A), the membrane is labeled with 0.1 mol% DPPE-Rh, whereas in (B), the membrane contains 0.5 mol% DPPE-NBD (false color green) and the vesicle encapsulates 2.5 μM SRB (red).

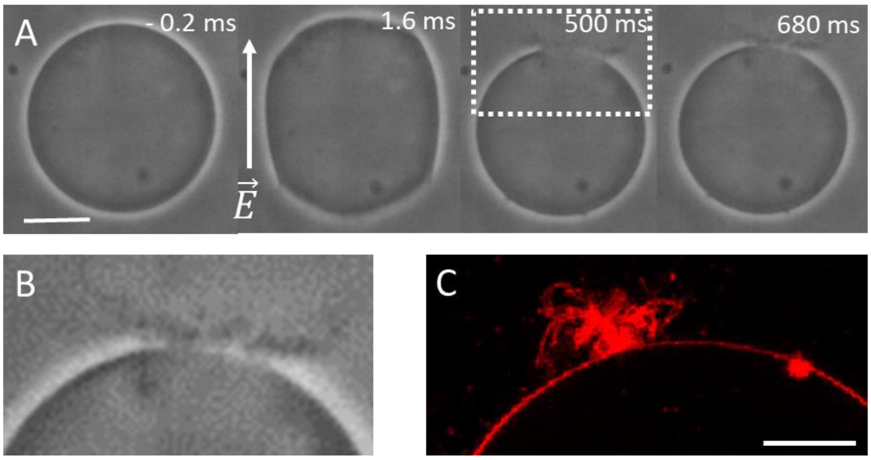

Figure S2. Partial bursting of charged (POPC:POPG; 1:1 mol) GUVs. (A) Phase contrast snapshots showing macropore closure associated with consumption of the membrane and conversion into tubular structures accompanied by a decrease in GUV size. The numbers correspond to time relative to the onset of the pulse. Electroporation in the presence of 1 mM NaCl. (B) Zoomed-in and enhanced image of the tubulated region in the last snapshot in panel (A, dashed square). (C) Confocal cross section of another GUV showing the tubules formed in the region where the macropore closed. The GUV in (C) contains 0.1 mol% DPPE-Rh. Scale bars correspond to 20 μm in (A) and 5 μm in (C).

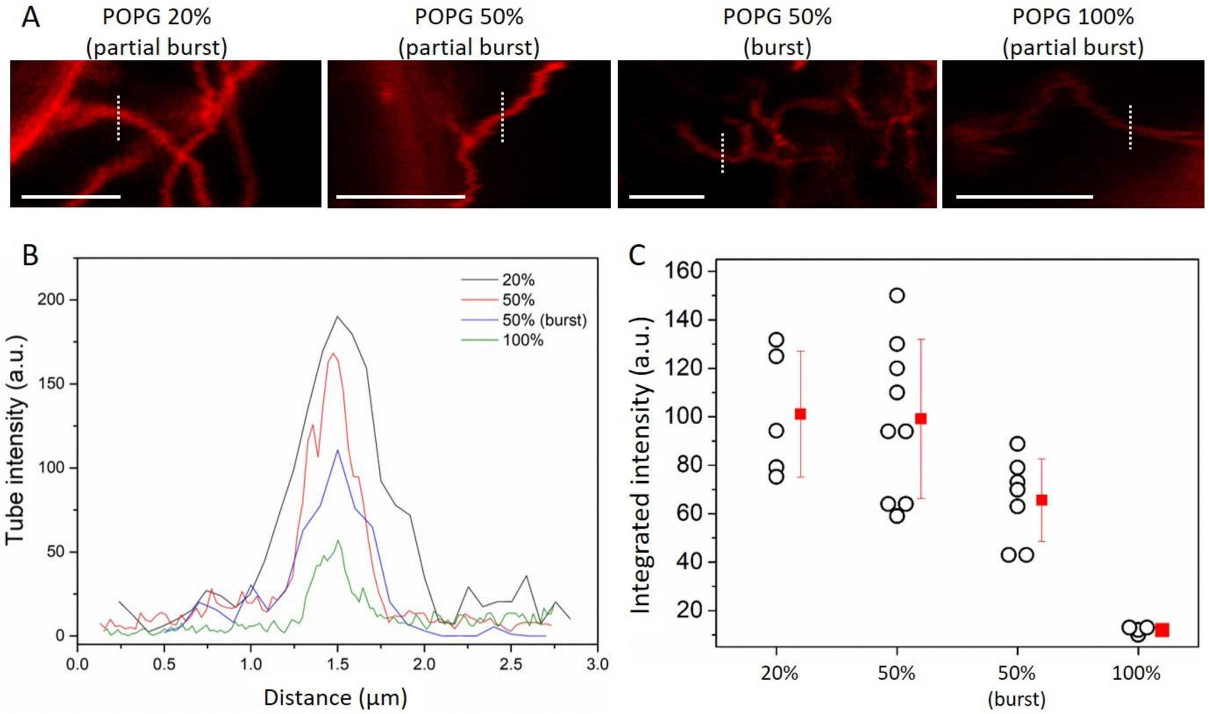

Figure S3. The diameter of lipid tubes formed on negative GUVs decreases with increasing membrane charge. To assess trends in the tube thickness, the tubes were imaged using identical microscope settings and a dye (DPPE-Rh, 0.1 mol%) that displays no curvature preference [3]. No variation in the fluorescence intensity of the vesicle containing different fractions of charged lipids were detected (before and after poration). (A) Images of lipid tubes formed after complete vesicle bursting (burst) or partial vesicle bursting (partial burst) where the tubes remain attached to the mother GUV. The POPG fraction in the membrane varies from 20 to 50 to 100 mol% from left to right. The images show weaker intensity associated with thinner tubes as the PG fraction increases. Scale bars: 5 µm. (B) Tube fluorescence intensity profiles from the vertical lines shown in (A). Line profiles were always taken in the same direction to minimize polarization issues. (C) Area-integrated peak intensities measured on tubes formed upon bursting (burst) or still attached to partially burst GUVs for increasing POPG mol%. Each open circle corresponds to a measurement on an individual tube. Measurements were performed on several GUVs per composition (occasionally, multiple measurements from independent tubes from the same GUV). A minimum of 3-4 GUVs per composition were studied. Mean and standard deviation are also shown (red). The decrease in intensity is associated with decreased tube diameter.

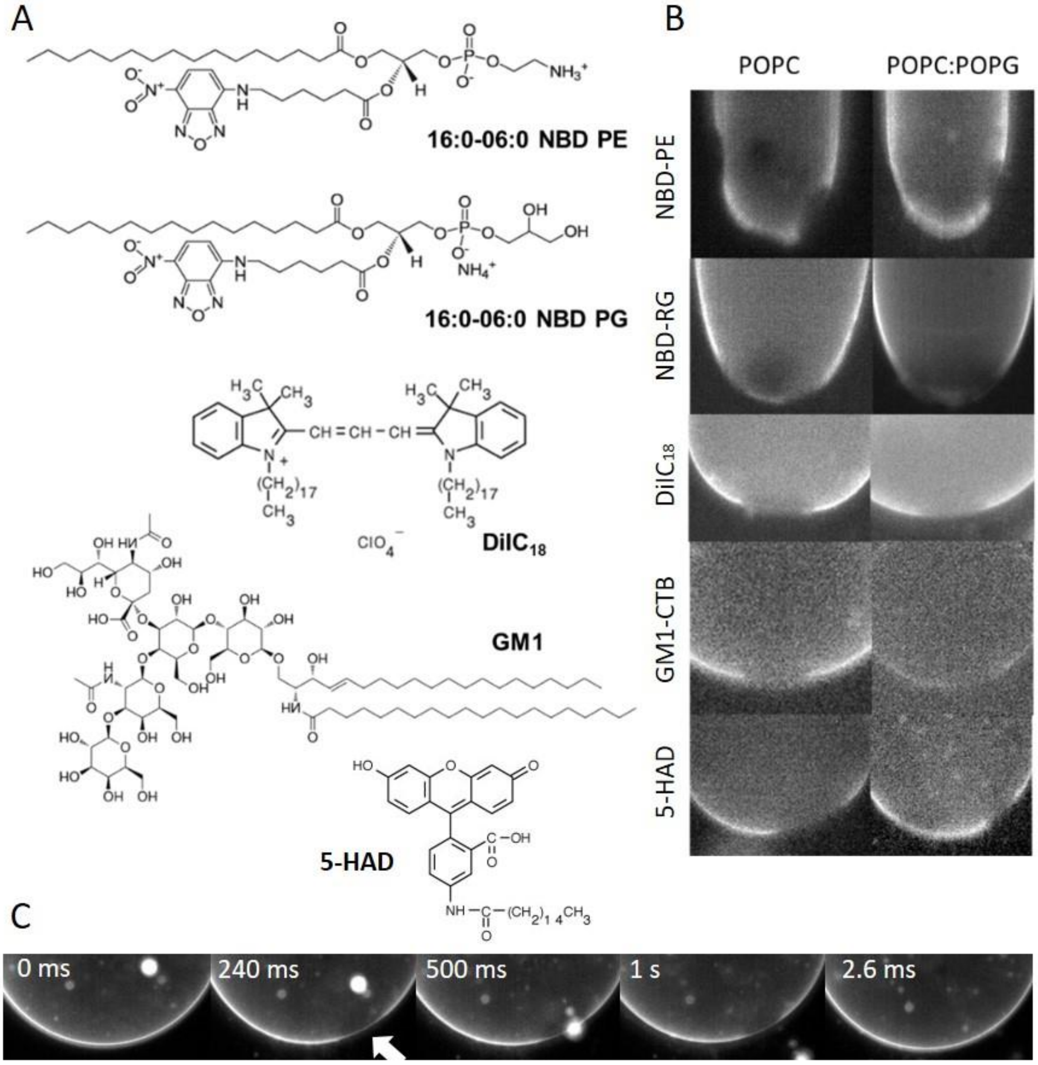

Figure S4. Composition in the pore rim vicinity is similar to that of regions of intact membranes as viewed with fluorescence markers. (A) Chemical structure of (some of) the used fluorescent probes of different charge and molecular geometry. Their concentrations in the membrane was varied from 0.1 to 1 mol%. (B) Snapshots of pores in neutral (POPC) or negative (POPC:POPG) GUVs doped with different dyes: neither accumulation nor depletion of the probes of diverse biophysical properties can be detected. GUV sizes varied from 30 to 60 μm. The individual frame exposure time was 5 ms or 20 ms. (C) Snapshots of the closure of long-lasting macropore on a negative GUV. Note the exit of internal structures through the pore. The membrane is labeled with NBD-PG.

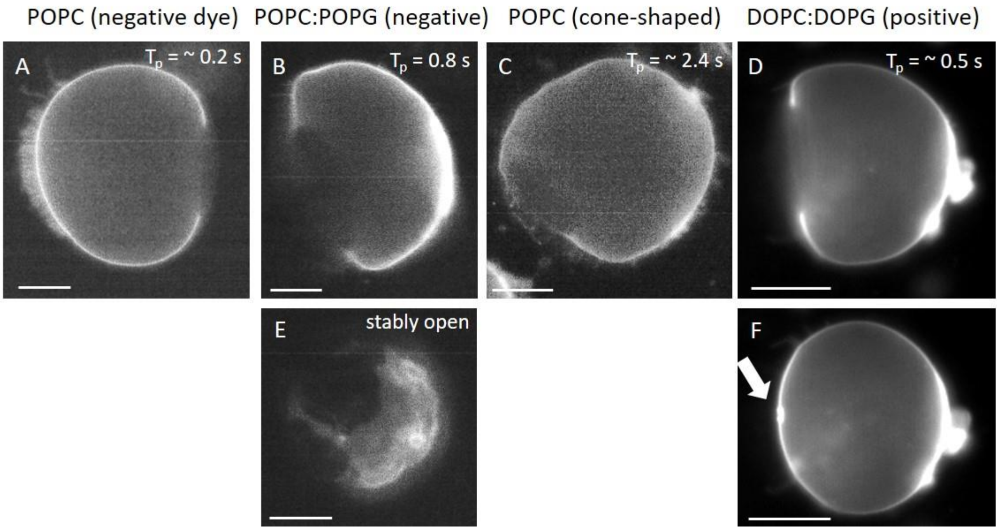

Figure S5. Lipid analogues do not accumulate in the rim of long-lasting pores. Following the protocol in [4], GUVs were immobilized in 1% (w/v) isotonic agarose gel to slow down pore closure and were exposed to a single DC pulse (4 kV/cm, 8 ms). Membrane compositions and dye properties are indicated above the images. The text in brackets describes the charge and shape of the fluorescent lipid analogues used. Negative: NBD-PG; cone-shaped: 5-HAD; positive: DPPE-Rh, see corresponding structures in Fig. S4A. (A-D) Representative behavior of electroporated GUVs of different composition. The numbers (top right) show the pore lifetime (T_p) for these specific vesicles. (E) POPC:POPG 1:1 GUV with stably open pores (T_p > 1 minute). (F) Small buds formed in the region where the macropore closed (arrow). The non-spherical GUV shapes result from the confining agarose gel mesh around the vesicles. Scale bars: 20 μm.

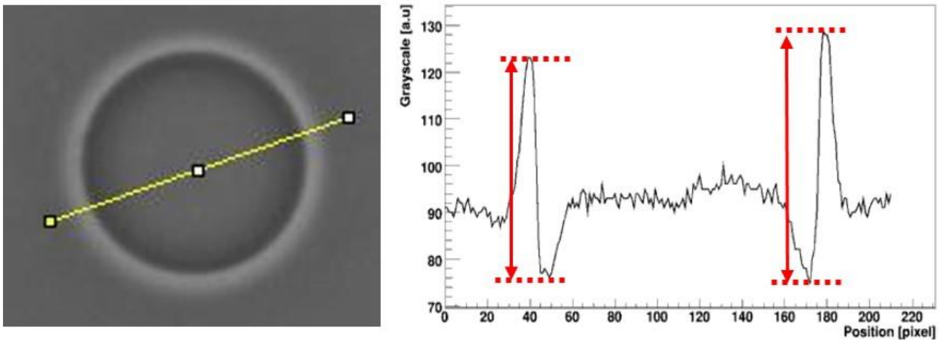

Figure S6. Measuring the optical contrast of a GUV imaged in phase contrast mode. A line is drawn across the vesicle (left) and the grayscale profile along this line is obtained (right). The optical contrast is defined as the average of the two heights indicated in red in the figure. The analyses were done with ImageJ.

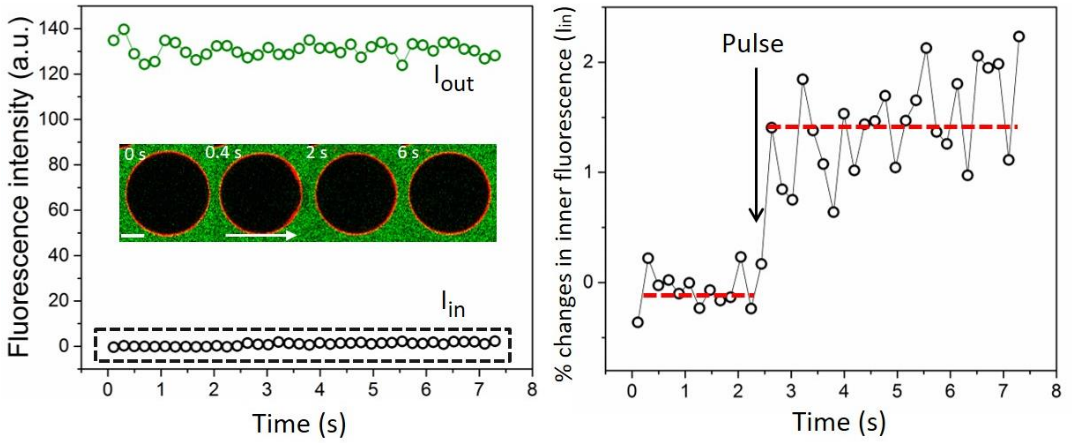

Figure S7. Only a small fraction of dyes enters the vesicles directly through macropores demonstrated from following the entry of a small external dye (calcein) upon GUV macroporation after which the vesicle reseals. (A) Electroporation of a negative GUV (0.1 mol% DPPE-Rh, red) in the presence of 5 μM calcein (green). The field direction is indicated with an arrow. The numbers correspond to the time after applying the pulse (3 kV/cm, 300 μs). Scale bar: 20 μm. Calcein fluorescence outside, I_{out}, and inside the GUV, I_{in}, is shown in the graph. (B) The increase in calcein fluorescence inside the GUV upon pulse application (arrow) is in the order of only ~ 1% (same data as in A).

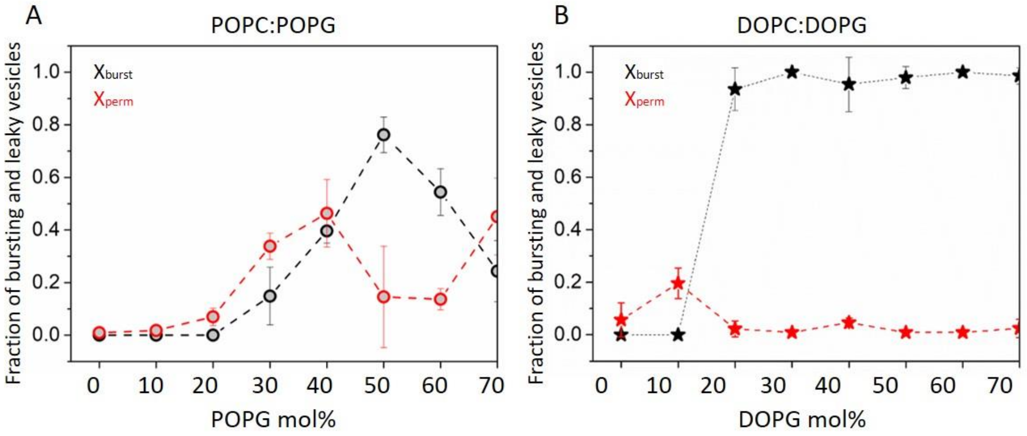

Figure S8. Fraction of bursting vesicles (black data), $X_{burst} = n_{burst}/n_{GUVs}$, and fraction of surviving vesicles which lose contrast (red data), $X_{perm} = n_{perm}/(n_{GUVs} - n_{burst})$, for increasing molar fractions of charged lipids. Panels (A) and (B) respectively show data for POPC/POPG and DOPC/DOPG vesicles with increasing fractions of the PG lipid. Average and standard deviation from a number of measurements on different vesicles (> 15) of a given composition are shown.

Text S2: Edge tension measurements

Pore dynamics in vesicles follows four well-defined stages; (i) quick opening, (ii) maximum size stage that is relatively stable, (iii) slow closure, limited by leak-out, and (iv) rapid closure as theoretically modeled in [5]. According to this model, the slow closure (third stage) of a circular pore of radius r in a GUV of radius R, can be directly related to the edge tension through $R^2 ln(r) = -(2\gamma/3\pi\eta)t + C$, where η is the medium viscosity ($\eta = 1.133\times10^{-3}$ Pa.s for the conditions studied here), t is time and C is a constant. R is assumed roughly constant during pore closure and measured after resealing of the macropore(s). The edge tension γ is directly calculated from the slope of the linear dependence of $R^2 ln(r)$ with time, see also Fig. S9. Vesicles with radius deviation larger than 5 % were discarded for

GUVs with resealing pores. The obtained mean edge tension values and standard deviations are given in Table S2. In the case of partial bursting where the vesicle size decreases as the pore closes (data in magenta in Fig. 4), the edge tension was evaluated considering the initial and final vesicle radius and averaged (error bars in Fig. 4 show the corresponding deviations resulting from vesicle size changes).

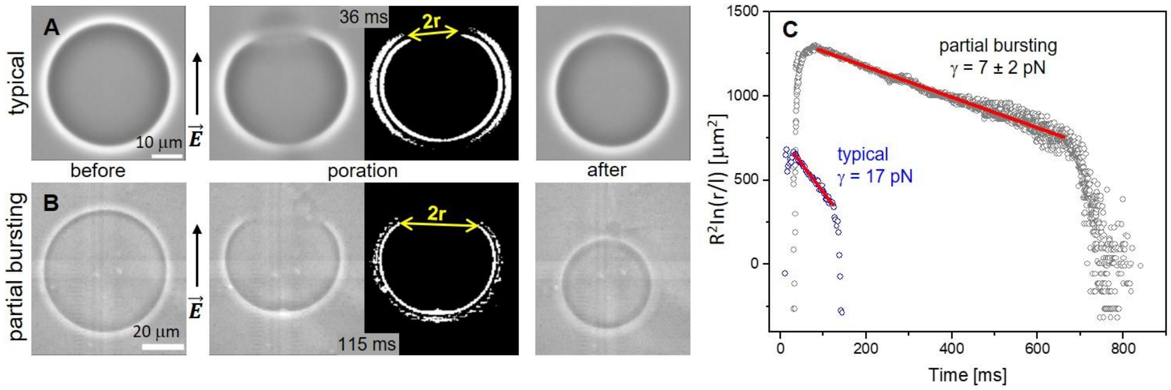

Figure S9. Pore edge tension measurement for negative POPC:POPG (1:1) GUVs. (A, B) Phase contrast snapshots of a GUV before, during and after pore closure, respectively, for typical pore closure (upper row) and partial bursting cases (lower row). The third snapshots in the rows are processed images (same as the second snapshots) used for measuring the pore radius r. The processing of the phase contrast images involves background subtraction and binarization as introduced in [6]. (C) Edge tension measurements showing the pore opening and closure and the fit for the slow closure stage (red lines) according to $R^2 ln(r) = -(2\gamma/3\pi\eta)t + C$ for typical (blue data) and partial bursting cases (black data). In the graph, the pore size r in µm is rescaled by l = 1 µm. Pore closure is significantly slowed down for the case of partial bursting. Accordingly, the edge tension extracted from the vesicle with a typical macropore is 17 pN, while the one obtained from the vesicle exhibiting partial bursting is only 7 ± 2 pN.

Table S2. Edge tension values (mean and standard deviation) obtained for different membrane compositions. For every condition (i.e. composition), various samples from one or two GUV preparation were examined.

Composition	Additives	Number of GUVs	γ (pN)
POPC	0.5 mM NaCl and 0.2 mM EDTA	8	39.5 ± 5.2
POPC with 8 mol% POPG	0.5 mM NaCl and 0.2 mM EDTA	16	40.8 ± 5.1
POPC with 16 mol% POPG	0.5 mM NaCl and 0.2 mM EDTA	16	39.5 ± 4.5
POPC with 24 mol% POPG	0.5 mM NaCl and 0.2 mM EDTA	7	40.8 ± 7.4
POPC with 30 mol% POPG	0.1 mM NaCl	16	40.8 ± 10.2
POPC with 35 mol% POPG	0.1 mM NaCl	10	39.9 ± 8.6
POPC with 42 mol% POPG	0.5 mM NaCl and 0.2 mM EDTA	4	31.3 ± 6.1
POPC with 50 mol% POPG	0.5 mM NaCl and 0.2 mM EDTA	8	23.4 ± 6.0
POPC with 50 mol% POPG	1 mM $CaCl_2$	12	62.1 ± 11.5

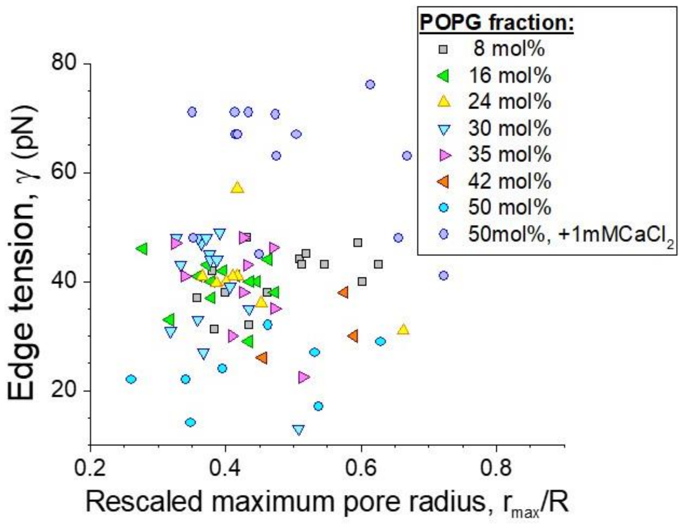

Figure S10. The measured edge tension does not depend on the maximum pore size r_{max} (here, rescaled by the GUV size, R) as measured for all POPC:POPG compositions in the absence and in the presence of 1 mM $CaCl_2$.

Text S3. Energetic considerations for pore expansion and closure

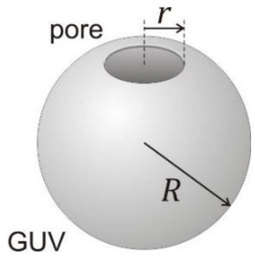

Figure S11. Sketch of a vesicle with a pore. The vesicle radius, R, and pore radius, r are indicated.

The energy of a porated vesicle of radius R is a sum of the Helfrich and the rim energy:

$$E = 2\kappa \int_A (M-m)^2 dA + \kappa_G \int_A dA/R^2 + 2\pi\gamma r, \quad \text{(S1)}$$

where κ is the membrane bending rigidity, $M = 1/R$ is the vesicle mean curvature, m is the membrane spontaneous curvature, κ_G is the Gaussian curvature modulus, r is the pore radius and γ is the edge tension. The integral in Eq. S1 is over the vesicle (nonporated) area $A = 2\pi R^2 \left[1 + \sqrt{1-(r/R)^2}\right]$.

For a pore to have the tendency to expand, as is the case in bursting PG-containing vesicles, the energy of the system should decrease, i.e.

$$dE/dr = -\frac{4\pi\kappa R(1/R-m)^2}{\sqrt{(R/r)^2-1}} + 2\pi\gamma < 0\,. \quad \text{(S2)}$$

Above we assumed that the vesicle radius remains constant, which for small changes in the pore radius is indeed the case. We see that the Gaussian curvature contribution (second term in Eq. S1) cancels out, which is understandable because the topology of a vesicle with expanding pore is preserved. Equation S2 implies that the edge tension term is dominated by curvature contributions.

PG-containing electroformed vesicles exhibit inward tubes [7] with diameter below the resolution of confocal imaging, $2R_{cyl}$ < 200nm. The associated spontaneous curvature for inward cylindrical tubes is $m \equiv -1/2R_{cyl}$ implying that $1/R \ll m$ and can be ignored. The inequality S2 can then be transformed into

$$m^{-2} < 2\kappa/\left(\gamma\sqrt{1/r^2 - 1/R^2}\right). \quad \text{(S3)}$$

Considering that the pore radius reached at the end of the pulse is of the order of 1 µm, i.e., $r \ll R$, the above expression roughly reduces to

$$|m|^{-1} < \sqrt{2\kappa r/\gamma}\ . \qquad \text{(S4)}$$

For 50 mol% PG membranes, we have γ = 23 pN (see data in Fig. 4B) and κ = 31 k_BT [8]. We thus obtain that the diameter of tubes stabilized by spontaneous curvature at which the vesicles tend to burst should be smaller than 105 nm. This result is consistent with the suboptical tube diameters we observe upon poration (Fig. S3). The predicted relatively high spontaneous curvature is plausibly generated by the asymmetric distribution of PG in the membrane resulting from the vesicles preparation method [7].

As a consistency check, for pure PC membranes where the pores tend to close, the energy of the systems should satisfy the opposite condition $dE/dr > 0$, i.e. it is energetically unfavorable for the pores to open. These single-component PC membranes are intrinsically symmetric and exhibit zero spontaneous curvature $m \approx 0$, which from Eq. S2 implies that $2\kappa/\gamma < R\sqrt{(R/r)^2 - 1}$. Taking for γ = 40 pN (Fig. 4B) and $\kappa \approx 2.1\times10^{-19}$ J [9], we see that this condition is indeed satisfied.

Supplementary movies

Movie S1. Bursting of a charged GUV upon electroporation observed under phase contrast microscopy. The GUV composed of POPC:POPG (1:1) in sucrose/glucose (in/out) solution was electroporated using a pulse of 3 kV/cm magnitude and 150 µs duration. The formed macropore expands, leading to a full collapse of the vesicle, leaving only tubular membrane fragments after bursting. The time stamps correspond to the initial observation time. The GUV was electroporated at time 34 ms. Vesicle diameter is around 25 µm.

Movie S2. Bursting of a charged GUV upon electroporation observed under epifluorescence microscopy. The GUV composed of POPC:POPG (1:1) prepared in sucrose/glucose (in/out) solution and labelled with 0.1 mol% DPPE-Rh was electroporated (3 kV/cm, 150 µs). The formed macropore expands by converting the nearly flat membranes into tubular lipids structures. The time stamps correspond to the initial observation time. The GUV was electroporated at time 17 ms. Vesicle diameter is around 30 µm. The much smaller vesicle seen at the end of the movie is another vesicle (already present in the chamber), which is not associated with the main GUV and which is not in focus in the beginning of the movie.

Movie S3. Bursting of a charged GUV upon electroporation observed under confocal microscopy. The GUV composed of POPC:POPG (1:1), labelled with 0.5 mol% DPPE-NBD (green), prepared in sucrose/glucose (in/out) solution and encapsulating 2.5 µM SRB (red) was electroporated (3 kV/cm, 150 µs). The GUV contains a couple of smaller vesicles inside. Bursting leads to the rapid release of the encapsulated content. These smaller vesicles do not burst because they are shielded by the outer vesicle and are too small for the transmembrane potential to reach the poration threshold (see Text S1). The time stamps correspond to the initial observation time. The GUV was electroporated at time 190 ms. Vesicle diameter is around 30 µm.

Movie S4. Long-lasting macropore in a charged GUV observed under epifluorescence microscopy. The GUV composed of POPC:POPG (1:1) containing 0.1% DPPE-Rh was electroporated (3 kV/cm, 150 µs) and the formed macropore remained opened for longer than 2 s. Such a long pore lifetime allowed the release of encapsulated smaller vesicles inside the GUV. Note that the fluorescence around the pore rim is homogenous, suggesting neither enrichment nor depletion of lipids. The time stamps correspond to the initial observation time. The GUV was electroporated at time 60 ms. Vesicle diameter is around 50-60 µm.

Movie S5. Electroporation of a neutral GUV (POPC) labelled with 0.5 mol% NBD-PG and immobilized in 1 wt% agarose. A single DC pulse (4 kV/cm, 8 ms) was applied at time ~1.1 s. The GUV was observed under epifluorescence microscopy. Vesicle size is around 40 µm.

Movie S6. Bursting of a charged GUV, POPC:POPG (1:1), induced by detergent and observed at high temporal resolution under phase contrast microscopy. A concentrated solution of Triton X-100 was added to the chamber (to a final concentration of 1 mM) containing POPC:POPG (1:1) GUVs. The movie is slowed down. The time stamps correspond to the initial observation time. The macropore appears at time 12.5 ms. The expansion of the bursting membrane is very fast and is completed in less than 3 s. Vesicle size is around 50-60 µm.

Movie S7. Bursting of a charged GUV, POPC:POPG (1:1), induced by detergent observed under epifluorescence microscopy. A concentrated solution of Triton X-100 was added to the chamber (to a final concentration of 1 mM) containing POPC:POPG (1:1) GUVs labelled with 0.1 mol% DPPE-Rh. At initial times, Triton X-100 leads to an increase in GUV area (the membranes look floppy). The numbers correspond to the initial observation time. The macropore appears at 3.7 s and leads to vesicle bursting with dynamics identical to that induced by electroporation. At later times, the membrane fragments formed after bursting are solubilized. Vesicle size is around 50-60 µm.

Movie S8. Formation of submicron-sized pores before vesicle bursting. A POPC:POPG (1:1) GUV labelled with 0.5 mol% DPPE-NBD and in the presence of 2.5 µM of SRB was incubated with Triton X-100 (1 mM final concentration) and observed under confocal microscopy. The movie is sped up. The vesicle becomes permeable to SRB (at ~ 40 s) before it bursts (at ~3 min). The macropore formation is too fast to be observed with confocal microscopy. Vesicle size is around 30 µm.

Movie S9. Macropores formed on charged GUVs are stabilized in the presence of $CaCl_2$. A POPC:POPG (1:1) GUV labelled with 0.1 mol% DPPE-Rh and in the presence of 3.5 mM $CaCl_2$ was incubated with Triton X-100 (1 mM final concentration) and observed under epifluorescence microscopy. The detergent-induced macropore remains open for many seconds until complete GUV solubilization, allowing the exit of encapsulated vesicles. The GUV becomes dimmer over time resulting from the combined effect of membrane solubilization and DPPE-Rh photobleaching. The initial vesicle size is around 50-60 µm.

References

[1] K. Kinosita, I. Ashikawa, N. Saita, H. Yoshimura, H. Itoh, K. Nagayama, A. Ikegami, *Biophysical Journal* **1988**, *53*, 1015-1019.

[2] a) T. Y. Tsong, *Biophysical Journal* **1991**, *60*, 297-306; b) J. C. Weaver, Y. A. Chizmadzhev, *Bioelectrochemistry and Bioenergetics* **1996**, *41*, 135-160; c) D. Needham, R. M. Hochmuth, *Biophysical Journal* **1989**, *55*, 1001-1009.

[3] a) Y. F. Baroji, L. B. Oddershede, S. N. Seyed Reihani, P. M. Bendix, *European Biophysics Journal* **2014**, *43*, 595-602; b) B. Sorre, A. Callan-Jones, J. B. Manneville, P. Nassoy, J. F. Joanny, J. Prost, B. Goud, P. Bassereau, *Proc. Natl. Acad. Sci. U. S. A.* **2009**, *106*, 5622-5626; c) A. Tian, T. Baumgart, *Biophysical Journal* **2009**, *96*, 2676-2688.

[4] R. B. Lira, J. Steinkühler, R. L. Knorr, R. Dimova, K. A. Riske, *Sci Rep* **2016**, *6*, 25254.

[5] a) F. Brochard-Wyart, P. G. de Gennes, O. Sandre, *Physica A* **2000**, *278*, 32-51; b) R. Ryham, I. Berezovik, F. S. Cohen, *Biophysical Journal* **2011**, *101*, 2929-2938.

[6] T. Portet, R. Dimova, *Biophysical Journal* **2010**, *99*, 3264-3273.

[7] J. Steinkühler, P. De Tillieux, R. L. Knorr, R. Lipowsky, R. Dimova, *Sci Rep* **2018**, *8*, 11838.

[8] H. A. Faizi, S. L. Frey, J. Steinkühler, R. Dimova, P. M. Vlahovska, *Soft Matter* **2019**, *15*, 6006-6013.

[9] R. Dimova, *Advances in Colloid and Interface Science* **2014**, *208*, 225-234.
